# Preliminary evidence of prolonged timing effects of theta-burst stimulation in the reading system

**DOI:** 10.3389/fnhum.2023.1227194

**Published:** 2023-08-29

**Authors:** Rachael M. Harrington, Lisa C. Krishnamurthy, Alexandra Ossowski, Mykayla Jeter, Adriane Davis, Ewelina Bledniak, Ashley L. Ware, Robin Morris, C. Nikki Arrington

**Affiliations:** ^1^Center for Research on the Challenges of Acquiring Language and Literacy, Georgia State University, Atlanta, GA, United States; ^2^Department of Communication Sciences and Disorders, Georgia State University, Atlanta, GA, United States; ^3^Center for Advanced Brain Imaging, Georgia State University, Atlanta, GA, United States; ^4^Center for Visual and Neurocognitive Rehabilitation, Atlanta VA Healthcare System, Decatur, GA, United States; ^5^Center for Translational Research in Neuroimaging and Data Science, Georgia State University, Atlanta, GA, United States; ^6^Department of Physics and Astronomy, Georgia State University, Atlanta, GA, United States; ^7^Department of Radiology and Imaging Sciences, Emory University, Atlanta, GA, United States; ^8^Department of Psychology, Georgia State University, Atlanta, GA, United States; ^9^Department of Neurology, University of Utah, Salt Lake City, UT, United States

**Keywords:** reading, transcranial magnetic stimulation, theta-burst stimulation, Neuromodulation, repetitive transcranial magnetic stimulation, reading facilitation

## Abstract

Theta-burst stimulation (TBS) is a repetitive transcranial magnetic stimulation technique that can be used to upregulate or downregulate different brain regions. However, the timing of its effects and the differing effects of continuous TBS (cTBS) versus intermittent TBS (iTBS) in the reading system have not been explored. This study assessed how stimulation type and post-stimulation timing affected change in performance during a phonological discrimination and sight word recognition task after stimulation of supramarginal gyrus (SMG). Fourteen right-handed young adults (age 18–27 years; 44% male) were block-randomized to receive either iTBS or cTBS to the supramarginal gyrus. Participants then performed a pseudoword discrimination task and an orthographic awareness task (behavioral control) at four different time points and change in reaction time compared to baseline was measured from each time point. There was no effect of stimulation type on change in reaction time [*t*(16) = −0.2, *p* = 0.9], suggesting that both types of TBS caused similar effects. Percent change in reaction time decreased over time in the pseudoword task [*t*(50) = −5.9, *p* < 0.001], indicating faster pseudoword processing speed with better performance 60–70 min after stimulation. In contrast, no change was demonstrated over time for the behavioral control task [*t*(43) = −0.6, *p* = 0.6], suggesting that the change over time seen in the test condition was not a learning effect. These findings provide insight into the effects of TBS on the reading system and can guide future study designs.

## 1. Introduction

Neural plasticity is induced and amplified by increased neuronal activity (Kleim and Jones, 2008; Magee and Grienberger, 2020). Early studies of plasticity in the hippocampus showed that synaptic connectivity between neurons increased following trains of high-frequency electrical stimulation and that the connectivity decreased following trains of low-frequency stimulation (Hebb, 2005). Transcranial magnetic stimulation (TMS) has been used to non-invasively replicate these principles in humans (Thickbroom, 2007). TMS causes a brief magnetic pulse at the scalp that induces an electrical current in the targeted, underlying cortical brain region (Harris-Love, 2012). When done repetitively (rTMS), at high or low frequencies, stimulation results in increases or decreases in cortical excitability which affect behavior, mimicking the effects of long-term potentiation or depression (Rossini et al., 2015).

One form of rTMS, theta-burst stimulation (TBS) performs this repetitive stimulation in 50 Hz “bursts,” decreasing the needed time for stimulation and making it a more tolerable methodology for human studies (Huang et al., 2005). TBS uses a rapid train of single TMS pulses to change the state of the underlying cortical excitability (Bestmann and Krakauer, 2015; Suppa et al., 2016). In the motor system, continuous TBS (cTBS) depresses the amplitude of motor evoked potentials (MEPs), while intermittent TBS (iTBS) increases the amplitude of MEPs (Suppa et al., 2016). This broadly suggests that iTBS is excitatory and that cTBS is inhibitory (Suppa et al., 2016). The literature also suggests differing effects of timing between each type of TBS. On average, cTBS has decreased MEP amplitude for up to 50 min and iTBS has increased MEP amplitude for an average of 60 min (Wischnewski and Schutter, 2015). However, the greatest change in MEP amplitude occurs 18–33 min post-stimulation with iTBS or 5 min after cTBS (Hinder et al., 2014; Vernet et al., 2014). These principles of the time course of TBS have not been examined in more complex cognitive networks, such as the reading network.

The reading network is inherently more complex than the network required to produce an MEP. Despite this, the effects of TBS on the reading network have thus far been assumed to be on time scales that are similar to the motor system (Arrington et al., 2022). Stimulation of the motor system results in an approximate 1:1 relationship between stimulation of cortical neurons and production of an MEP. Two motor neurons (corticospinal tract and alpha motor neuron) are all that is needed to evoke the desired behavioral response (Brasil-Neto et al., 2009). Stimulation of a more complex cognitive system, like the reading network, is theoretically less straightforward. The reading network integrates visuospatial pattern-recognition, language, attention, memory, executive function, and articulatory motor networks. Therefore, the more complex neural architecture and number of networks recruited during reading may reflect a less linear relationship between stimulation of cortical neurons and reading output than the system needed to produce an isolated MEP (Krishnamurthy et al., 2019). The reading network is not one system, but the coordination of three distinct systems supporting underlying cognitive outcomes (Pugh et al., 1996). The coordination of these networks is more effortful and the spread of stimulation likely requires a longer time course, leading the effects of TBS to potentially peak later and last longer in the reading network than the time course reported in the motor literature. In fact, a review found that effects of TBS are much more variable when affecting complex cognitive systems than motor and somatosensory function (Demeter, 2016). Understanding the time course effects of TBS on complex cognitive systems is critical for evaluating the effects of stimulation on cognitive functions such as reading. It also has implications for the use of TBS in treatment paradigms.

According to conventional models of reading, the reading system is comprised of three primary subnetworks: dorsal, ventral, and anterior (Pugh et al., 1996). The dorsal stream is classically thought to be involved in phonological decoding, or mapping sounds to visual representation, with a primary node in the supramarginal gyrus (SMG) (Pugh et al., 2001). The ventral stream facilitates reading fluency and sight word recognition, with a primary node in middle temporal gyrus (Pugh et al., 2001). The anterior stream is involved in articulatory recoding with a primary node in pars triangularis (Pugh et al., 2001). Tasks that involve phonological discrimination have been shown to engage the dorsal stream while tasks that involve sight word recognition have been shown to engage the ventral stream (Pugh et al., 2001). The SMG sits within the dorsal stream and is expected to influence phonological decoding but not sight word recognition allowing these two distinct components of reading to be both functionally and structurally differentiated. Here, we use this model to examine the facilitation and timing principles of TBS that have yet to be fully understood in the complex reading system. Specifically, this study assessed change in phonological discrimination and sight word recognition reaction time post-stimulation to SMG. We aimed to address the following research questions within the context of well-studied reading networks:

1. Do cTBS and iTBS to SMG lead to differential effects on change in post-stimulation reaction time during a phonological decoding task? We hypothesize that cTBS will inhibit and iTBS will facilitate reading behavior as demonstrated by a change in reaction time during a phonological discrimination task.
2. What are the effects of time post-stimulation on change in reading behavior after stimulation of SMG? We expected a prolonged timing effect post-stimulation on reading behavior.

## 2. Methods

### 2.1. Participants

Adults age 18–30 (22.2 (4) years) were recruited via fliers posted at universities around the Greater Atlanta area as part of a neuroimaging study of reading networks in typical readers. The joint Georgia State University/Georgia Institute of Technology Center for Advanced Brain Imaging Institutional Review Board approved the protocol and procedures. Written consent was obtained from each participant.

Participants met the following inclusion criteria: between the ages of 18–60 years, a native speaker of English, right-handed, have a Full Scale IQ score of low average or higher (FSIQ-2=>80) based on the 2-subtest version of the Wechsler Abbreviated Scale of Intelligence [WASI-2 (Wechsler, 2011)], and have reading performance within age expected norms (=>90) based on the Woodcock Johnson-3 [WJ-III (Schrank et al., 2014)] Broad and Basic Reading Scores and the Test of Word Reading Efficiency [TOWRE-2 (Torgesen et al., 2012)] Sight Word Efficiency (SWE) and Phonemic Decoding Efficiency (PDE) measures (Table 1). These tests are normed for the age range of participants and are well-established measures of reading ability (Wendling et al., 2007). Participants were excluded if they did not meet the criteria for TMS or MRI safety (e.g., history of seizure, metal in the body), or had history of a severe neurological or psychological disorder (Rossi et al., 2009). MRI contraindications were necessary as participants underwent two MRI scans (one for neuronavigation and one for post-stimulation data collection) as part of a larger parent study.

**TABLE 1:** Demographic data.

Demographic data		
	iTBS condition	cTBS condition
Age	23.4 (4.8)	20.75 (3.0)
Gender (F/M)	5/5	5/3
Race (A/B/W)	1/5/4	0/4/4
Years of education	15.7 (2.7)	14.6 (1.9)
WJ-3 Broad score	110.4 (10.5)	113.6 (8.6)
TOWRE-2 SWE	112.5 (10.7)	109.9 (12.6)
TOWRE-2 PDE	107.1 (7.3)	108.4 (6.4)

### 2.2. Experimental paradigm

#### 2.2.1. Screening, randomization, and pre-testing

Relevant demographic and reading history were collected at a pre-screening appointment. Inclusion criterion tests were completed at that time (see above).

Eligible participants were then block-randomized into two potential stimulation conditions (cTBS or iTBS). There were four participants who completed both conditions in accordance with their randomization in the parent study. A minimum wash-out period (range = 12 to 63 days) was maintained to minimize carryover effects between stimulation sessions. Participants also completed a T1-weighted MRI scan (TR: 2,250 ms, voxel size: 1.0 mm × 1.0 mm × 1.0 mm) on a 3 Tesla Siemens Tim Trio scanner at the GSU/GA Tech Center for Advanced Brain Imaging, which was used for neuronavigation during stimulation.

#### 2.2.2. Baseline testing

Participants completed two baseline computerized reading tasks during the initial MRI scan.

Pseudoword discrimination (PD) task: The visual pseudoword discrimination task is a computerized forced-choice, reaction time assessment of phonological processing. It measures the speed and accuracy of participants’ ability to phonetically decode a string of letters. This task is simple with high accuracy and has proven highly sensitive to individual differences in phonological decoding skills (Olson et al., 1994). Participants are presented with a letter string on the screen and are asked to judge via button press whether the letter string can be pronounced like a “real word” (e.g., “roze”) or not (e.g., “heaf”). Pronounceable and unpronounceable pseudowords are intermixed pseudo-randomly and response times and accuracy rates are recorded on a total of 50 trials. The median response time and accuracy rate across correct trials is calculated for each participant. This task is expected to activate the dorsal stream and engage SMG. This task served as our primary outcome measure.

Orthographic awareness (OA) task: This computerized forced-choice, reaction time assessment measures how quickly and automatically participants can identify valid orthographic patterns. This well-validated and well-replicated experimental task is used to measure the speed at which participants can identify whether or not a string of letters represents a correctly spelled real word (Olson et al., 1994). Participants see a letter string on the screen that either represents a correctly spelled real word (e.g., “flute”) or an incorrectly spelled word (e.g., “roze”). They are asked to judge via button press whether the letter string is a “real word” or “not a real word.” Correctly and incorrectly spelled words are intermixed pseudo-randomly and response times and accuracy rates are recorded on a total of 32 trials. The median response time across correct trials is calculated for each participant. This task is expected to engage the ventral stream. This task is not expected to engage the dorsal stream and therefore should not be impacted by stimulation of SMG, serving as our behavioral control measure.

#### 2.2.3. TMS stimulation

During TMS stimulation, participants were seated in a comfortable chair with legs uncrossed. A TMS cap was placed on the participant and tied securely under the chin. The starting point for establishing the motor thresholding hotspot was identified as the halfway point between the nasion and inion and 1/3 of the length between the center point (vertex) and the tragus of the left ear. Primary motor cortex (motor hotspot) was identified in this area using visual confirmation of muscle activation in the relaxed first dorseus interosseus (FDI) muscle following single-pulse TMS from a figure-of-eight coil (C-B60) connected to a MagVenture MagPro X1100 Magnetic Stimulator. Once the hotspot was identified, participants were asked to raise their right arm at a 90-degree angle in front of their torso and contract their right hand into an “OK” symbol. Active motor threshold (AMT), or the minimum stimulator output required to cause muscle contracture in the actively contracted hand, was determined using the adaptive parameter estimation by sequential testing (PEST) paradigm from the Medical University of South Carolina and visual confirmation of muscle contraction in the FDI (Awiszus and Borckardt, 2011).

Once AMT was identified, fiducial sensors were placed on the participant’s head and registered to their skull. Using Localite Neuronavigation software, SMG (apex of ascending ramus of superior parietal gyrus) was anatomically defined on the participant’s T1-weighted MRI for neuronavigation (Jung et al., 2016). Theta-burst conditions were delivered at 80% of the individual-determined AMT with a static cooled figure-of-eight magnetic coil (MCF-B65). Excitatory iTBS consisted of a series of 20 sets of 3-pulse bursts given at 50 Hz repeated every 200 ms for a total of 600 pulses (Huang et al., 2005). Inhibitory cTBS consisted of 40 s of uninterrupted 3-pulse bursts. One session of inhibitory stimulation delivers a total of 600 pulses (Huang et al., 2005). Participants were asked to read silently TOWRE-2 SWE Form-A during stimulation to activate the reading network.

#### 2.2.4. Post-testing

Immediately following stimulation, a timer was started and participants completed a second MRI scan that included repeated testing on the PD and OA tasks. These tasks were performed at four time points post-stimulation: (i) ∼15 min post (timepoint A), (ii) ∼30 min post (timepoint B), (iii) ∼60 min post (timepoint C), and (iv) ∼75 min (timepoint D) (see Table 2).

**TABLE 2:** Descriptive statistics for percent change in reaction time in the phonological decoding (PD) and orthographic awareness (OA) conditions after intermittent TBS (iTBS) and continuous TBS (cTBS).

	Timepoint A (∼15 min)	Timepoint B (∼30 min)	Timepoint C (∼60 min)	Timepoint D (∼70 min)
**Change in reaction time in the PD condition**				
Percent change post-iTBS	−16% (18%)	−18% (19%)	−22% (22%)	−25% (21%)
Percent change post-cTBS	−11% (21%)	−16% (19%)	−22% (17%)	−26% (17%)
**Change in reaction time in the OA condition**				
Percent change post-iTBS	−11% (13%)	−10% (11%)	−13% (13%)	−11% (18%)
Percent change post-cTBS	−10% (9%)	−12% (12%)	−19% (11%)	−8% (22%)

### 2.3. Data analysis

Participant responses during the pre- and post-stimulation PD and OA tasks were binarized by accuracy (i.e., correct versus incorrect). The median reaction time for each task iteration was calculated based on the reaction time of correct responses only (Willems et al., 2011). Post-stimulation reaction time at each time point was normalized against pre-stimulation reaction time by calculating the percent change as follows:


(1) %Δi=100*(x2-x1x1)

Where %△ is the percent change in reaction time at time point *I* (i.e., A, B, C, or D), x_1_ is the median reaction time from the baseline (pre-stimulation) session, and x_2_ is the post-stimulation median reaction time from time point *i*.

### 2.4. Statistical analysis

#### 2.4.1. Descriptive statistics

Descriptive statistics (mean and standard deviation) were calculated for demographic variables, timing data, and performance for participants in each stimulus condition (cTBS or iTBS). Comparisons were conducted using chi-square and *t*-test techniques in R v4.1.3.

#### 2.4.2. Linear mixed-effects modeling

Multiple linear mixed-effects models were computed using the *lmer* function from the *lme4* package in R (Bates et al., 2015). Comparison values were calculated using *lmerTest* (Kuznetsova et al., 2017). %Δ in either the OA or PD tasks was included as the dependent variable with stimulation type (cTBS or iTBS) and/or time (minutes) post-stimulation entered as fixed effects and participant as a random effect (Pinheiro et al., 2022). Including participant as a random effect accounted for repeated measures in some subjects. To determine the significance of type and time post-stimulation, likelihood ratio tests (LRT) were used to compare main effects models of stimulation type:


lmer(%△∼Stimulationtype+(1|Participant))

Or time post-stimulation:


lmer(%△∼TimePoststimulation+(1|Participant))

Against a model with both fixed effects:


lmerTest::lmer(%△∼Stimulationtype*TimePoststimulation

+(1|Participant))

And an intercept only model:


lmer(%△∼1+(1|Participant))

We conducted an analysis to estimate Cohen’s d as a measure of effect size using the *t_to_d* package from the *effectsize* package (Ben-Shachar et al., 2020).

#### 2.4.3. *Post-hoc* analysis

Pairwise comparisons of means were conducted using Tukey contrasts to examine differences in %Δ_PD_ and %Δ_OA_ across the four timepoint categories within the model. The estimates, standard errors (SE), z-values, and *p*-values are reported. The significance level was set at α = 0.05. Adjusted *p*-values were reported using the false discovery rate (FDR) method.

## 3. Results

### 3.1. Demographics

Fourteen participants were included in the analysis. Of those, 4 participated in both stimulation conditions, separated by a washout period, for a total of 18 datasets between the two conditions. For each participant, repeated measures were obtained with four time points for both the PD and OA tasks. Eight participants were included in the cTBS condition and 10 in the iTBS condition.

Participants who completed cTBS did not differ from those who completed iTBS in terms of age, gender, race, or years of education (see Table 1).

### 3.2. Effect of type of stimulation

The results of the LRT show that the addition of type of stimulation did not improve the fit of the model above the intercept in either the PD [χ^2^(1) = 0.03, *p* = 0.9] or OA conditions [χ^2^(1) = 0.06, *p* = 0.8] indicating that type of stimulation did not affect Δ%_PD_ or Δ%_OA_ (see Tables 2–5).

**TABLE 3:** Mixed-linear effects modeling and *post-hoc* analysis for change in reaction time in the phonological decoding (PD) condition.

Comparative model values in the PD condition						
Predictors	Estimates	CI	df	*p*-value	*d*	CI
**Main effect of type of stimulation**						
Type of stimulation	−1.3%	−19%, 16%	16	0.9	−0.1	−1.06, 0.91
**Main effect of time post-stimulation**						
Time post-stimulation	−0.2%	−0.3%, −0.1%	50	− 0.001***	−1.7	−2.30, −1.01
**Interaction effect of type of stimulation and time post-stimulation**						
Interaction effect	0.1%	−0.05%, 0.2%	49	0.2	0.4	−0.22, 0.91
**Intercept-only**						
Intercept	−20%	−28%, −11%	17	− 0.001***		
**Pairwise-comparisons of percent change in the PD condition between timepoints**						
**Comparison**	**Estimate**		**Std. error**	**z-value**		***p*-value**
B-A	14%		7%	2.0		0.04*
C-A	35%		17%	2.1		0.04*
D-A	44%		22%	2.0		0.04*
C-B	20%		10%	2.0		0.04*
D-B	30%		15%	2.0		0.04*
D-C	10%		5%	1.8		0.08

Letters in comparison column refer to timepoints. Timepoint A = ∼15 min, Timepoint B = ∼30 min, Timepoint C = ∼60 min, and Timepoint D ∼70 min. Each pairwise comparison is between the percent change value at the listed timepoints. Estimate of random effects (participant) = 0.3; significance levels are denoted by asterisks (*). *p < 0.05; ***p < 0.001.

**TABLE 4:** Mixed-linear effects modeling and *post-hoc* analysis for change in reaction time in the OA condition.

Comparative model values in the OA condition						
Predictors	Estimates	CI	df	*p*-value	*d*	CI
**Main effect of type of stimulation**						
Type of stimulation	1.5%	−22%, 15%	13	0.8	0.1	−0.96, 1.22
**Main effect of time post-stimulation**						
Time post-stimulation	−0.3%	−0.1%, 0.1%	43	0.6	−0.2	−0.77, 0.43
**Interaction effect of type of stimulation and time post-stimulation**						
Interaction effect	0.02%	−0.2%, 0.2%	42	0.9	0.1	−0.55, 0.66
**Intercept-only**						
Intercept	−11.8%	−18%, −5%	14	0.002**		
**Pairwise-comparisons of percent change in the OA condition between timepoints**						
**Comparison**	**Estimate**		**Std. error**	**z-value**		***p*-value**
B-A	1%		9%	0.1		0.9
C-A	−2%		21%	−0.1		0.9
D-A	7%		28%	0.3		0.9
C-B	−3%		13%	−0.2		0.9
D-B	5%		19%	0.3		0.9
D-C	9%		7%	1.2		0.9

Letters in comparison column refer to timepoints. Timepoint A = ∼15 min, Timepoint B = ∼30 min, Timepoint C = ∼60 min, and Timepoint D ∼70 min. Each pairwise comparison is between the percent change value at the listed timepoints. Estimate of random effects (participant) = 0.3; significance levels are denoted by asterisks (*). **p < 0.05.

**TABLE 5:** Results of likelihood ratio test (LRT) for the phonological decoding (PD) and orthographic awareness (OA) conditions.

	AIC	BIC	LL	Deviance	*x* ^2^	df	*p*-value
**LRT PD condition**							
Intercept	−91.6	−84.8	48.7	−97.6			
Main effect of time post-stimulation	−116.5	−107.5	62	−124.5	26.9	1	< 0.0001***
Main effect of type of stimulation	−89.6	−80.7	48.8	−97.6	0.1	1	0.8
Time × type	−114.5	−103.3	62.3	−124.5	26.9	1	< 0.0001***
**LRT OA condition**							
Intercept	−85.9	−79.7	45.9	−91.9			
Main effect of time post-stimulation	−84.2	−75.9	46.1	−92.2	0.3	1	0.6
Main effect of type of stimulation	−83.9	−75.7	45.9	−91.9	0.1	1	0.8
Time × type	−82.3	−71.9	46.1	−92.3	0.4	2	0.8

LRT, likelihood ratio test; PD, phonological decoding task; OA, orthographic awareness task; AIC, Akaike information criterion; BIC: Bayesian information criterion; LL: log-likelihood. Significance levels are denoted by asterisks (*). ***p < 0.001.

### 3.3. Effect of time post-stimulation

The LRT results indicated that the main effect model that included time post-stimulation was the best fitting model for Δ%_PD_ [χ^2^(1) = 26.9, *p* < 0.0001] (see Tables 2, 3, 5). These results show that time post-stimulation has a significant effect on Δ%_PD_. A large effect size (*d* = −1.7, CI = −2.30, −1.01) was calculated for this predictor, indicating a large and significant effect on the response variable.

Time post-stimulation did not improve the fit of the model above the intercept in the OA condition [χ^2^(1) = 0.3, *p* = 0.6]. These results show that the effect of time post-stimulation seen in the PD condition was not a learning effect (see Tables 2, 4, 5).

### 3.4. Effect of type of stimulation and time post-stimulation

The interaction of type of stimulation and time post-stimulation did not improve the fit of the model above the intercept in the OA condition [χ^2^(1) = 0.4, *p* = 0.8]. Including both effects did improve the fit of the model above the intercept in the PD condition [χ^2^(1) = 26.9, *p* < 0.0001], however, this appears to be primarily driven by the effect of time, not by the interaction effect (see Tables 3–5).

### 3.5. *Post-hoc* analysis

Pairwise comparisons were conducted to examine percent change in the PD condition between the four different timepoints (∼15, 30, 60 and 70 min after stimulation). The comparisons revealed significant differences in all pairs except for the comparison between timepoints C and D, which did not reach statistical significance (Table 3).

No significant differences between timepoints were found in the OA condition (Table 4).

## 4. Discussion

The goal of this study was to provide preliminary evidence about the facilitation type and timing effects of TBS in the reading network. In 14 young adults, we found that there were no differences in performance (i.e., percent change in reaction time) between cTBS and iTBS for our active condition or our behavioral control tasks. Thus, the facilitation and inhibition effects of TBS are not seen in behavioral reading measures as has been previously reported in the motor system. Instead, within our sample, both types of stimulation were associated with near equal facilitation or speeding of phonological processing. In contrast, the effect of TBS was significantly influenced by time post-stimulation, with the greatest effect demonstrated 60–70 min, and exceeding 80 min, post-stimulation. We did not see this effect of time in our behavioral control condition. This indicates that, in this study, a timing effect exists outside of a repeated exposure learning effect or task habituation. When considering the neural bases of these findings, the SMG is situated in the dorsal stream and therefore stimulation of this area should have more of an effect on performance during PD than in OA, as is seen in our results.

Interestingly, there was no significant difference between facilitation of change in reaction time on the PD task following cTBS and iTBS. In the motor system, cTBS is thought to mimic long-term depression, leading to an inhibitory effect, while iTBS is thought to mimic long-term potentiation that causes an excitatory effect (Chung et al., 2016). There is evidence, however, that this effect is inconsistent. Previous studies have found that MEP amplitude was consistently affected by on-line, single-pulse stimulation. However, when TBS was performed prior to single-pulse stimulation, the MEP response was variable, and for individual participants, the elicited MEP responses could not be reliably reproduced across sessions (Wischnewski and Schutter, 2015). This previous study added to a growing body of evidence that cTBS can elicit an excitatory response within the motor system (Demeter, 2016; Ozdemir et al., 2021). In our sample, this excitatory response after cTBS was found in the reading system with both forms of TBS resulting in general facilitation of change in phonological processing speed in all participants. Inhibitory effects were not seen in any participant.

Several studies have suggested that the effects of TBS observed in the motor cortex might not necessarily apply to other cortical regions (Tupak et al., 2013; Tsang et al., 2014; Painter et al., 2015). There are limited studies available to contextualize the timing results found in this study within the cognitive system. One previous study found that trains of TBS applied during the retention phase of a working memory task increased response time more than trains of TBS applied during the retrieval phase, indicating that, at least for short bursts of stimulation, some time is required for TBS to have an effect (Luber et al., 2007). This difference, however, was over the course of seconds—not on the order of minutes as seen in our results. No other studies have reported the effects of time for TBS and cognition (Demeter, 2016). Several studies, however, have called for future work examining the facilitation and timing effects of TBS in cognitive tasks (Luber et al., 2007; Demeter, 2016).

There are several potential explanations for the discrepancy between response to iTBS and cTBS seen in this study and those seen in previous literature. The first is that the difference is dependent on the relative size and complexity of the networks (Pugh et al., 1996). The reading network is comprised of three streams working in parallel across all lobes of the brain while the network that is required for production of an MEP is relatively simple, in comparison (Pugh et al., 1996). In addition, cognitive tasks, such as reading, require the coordination of multiple brain regions which increases the computational likelihood of variability of neuronal response (Malins et al., 2018). Finally, cognitive tasks have more competition from neural noise which may also increase individual variability and play a role in the discrepancies seen in this study (Hancock et al., 2017). The competition of neural noise within the reading network and the individual variability has been demonstrated in imaging studies of reading impairment (Hancock et al., 2017; Malins et al., 2018; Arrington et al., 2019).

Many studies simply infer that a behavioral outcome is due to excitatory or inhibitory stimulation without neurophysiological measures to confirm that assertion (Ozdemir et al., 2021). Some studies, however, have gathered neurophysiological data along with cTBS outcomes. These studies have used cTBS to suppress neural activation or reduce functional connectivity and have had varying degrees of success in this effort (Ott et al., 2011; Valchev et al., 2015; Steel et al., 2016; Shang et al., 2020). This effect has been demonstrated in the semantic system, which is intertwined with the reading system (Jung and Lambon Ralph, 2016, 2021). It is worth noting that many of the studies that have more consistent results demonstrating inhibition after cTBS stimulation are performed in the motor system with stimulation of the primary motor cortex (Chung et al., 2016). Functional data analyses of the current sample is ongoing to provide neuroimaging data to add to these results.

The preliminary timing effects seen in this analysis have potential implications for future study design. As discussed previously, the available guidance on timing effects suggest windows of effectiveness up to 50 (cTBS) and 60 (iTBS) minutes post-stimulation with a peak effect after 18–33 min (iTBS) or 5 min (cTBS) post-stimulation (Hinder et al., 2014; Vernet et al., 2014; Wischnewski and Schutter, 2015). Our results suggest that in the more complex reading network, this window is much longer with a potentially much later peak. SMG is situated in the dorsal stream and therefore stimulation of this area should have more of an effect on performance during PD than in OA, as is seen in our results.

Understanding the timing of TBS effects is needed to design studies that target behavioral change or measure outcomes during the peak effect of stimulation. Inaccurate understanding of timing effects could decrease the effectiveness of TBS as an adjuvant to behavioral change techniques or lead to null or inaccurate results with outcome measures collected outside of an optimal range. This effect is particularly robust when you consider that these data were collected in a sample of typically developed readers. It is unusual to get a large effect from this population as there is often a ceiling effect for change in an otherwise well-established and automatized behavior. These results should be replicated in a larger sample size before firm conclusions about future study design are drawn. It would also be useful, in larger samples, to look at some of the known influences on response to TBS (genetic variability, developmental factors, age, cortical network activity, and neurotransmitter and receptor variation) to better understand what is driving the observed effects. We were unable to test for these potential causes of variability within this sample.

**FIGURE 1:**
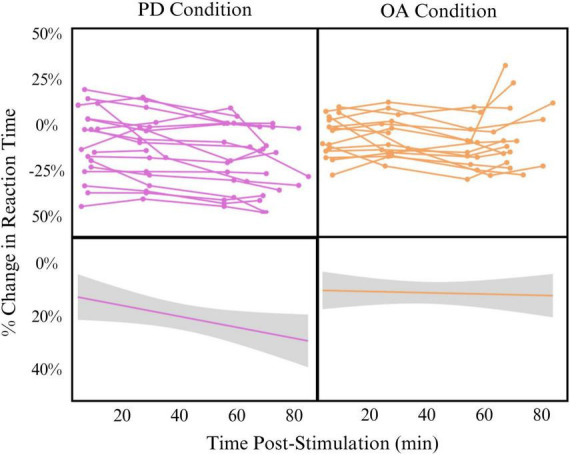
Effect of time post-stimulation on percent change in reaction time in phonological decoding (PD) **(left)** and orthographic awareness (OA) **(right)** conditions **Top:** individual spaghetti plots showing participant change in reaction time across time post-stimulation. **Bottom:** linear model of all participants change in reaction time across time post-stimulation (Change∼Time). Model is smoothed with standard error.

Despite promising implications, this study has several limitations that impact generalizability. Most importantly, the parent study was not initially designed to answer the question of timing and facilitation effects. As such, there is a range of time post-stimulation for each time point, and some overlap is observed in timepoints A to B and timepoints C to D. We have addressed this limitation by analyzing the data as a continuous-time variable via mixed-effects linear modeling instead of binned time points. Additionally, our control condition for learning effect was limited. We employed a behavioral control rather than sham TMS in this pilot sample. Though sham would have been preferred, our lab lacked sham capabilities during the period in which this data were collected. The effects of the behavioral control, however, are robust and show that the increased processing speed seen in the PD task after stimulation of the SMG is not solely a learning or habituation effect. In fact, it appears that the learning effect hovers between 10 and 15% while behavioral change occurs between 15 and 25% change in reaction time. Participants also performed the baseline and primary outcome measures while lying in the MRI scanner. Because this setting was consistent across baseline and outcome measure settings, we do not believe that this influenced the results. The sample size is relatively small, but repeated measures across participants help to address this limitation and Cohen’s d shows a very large effect of time in the PD condition. TBS is known to have wide inter-individual variability (Hinder et al., 2014). We controlled for this by including each participant in the mixed linear effects model as a random effect. We do not believe, however, that this variability was a contributing factor to our results, as demonstrated by individual spaghetti plots as shown in Figure 1.

For future analyses, we strive to further understand the neurobiology that supports TBS-induced changes by pairing the behavioral information reported in this study with gathered functional imaging data post-stimulation. We plan to extend this study design to a population of participants with aphasia, alexia, and dyslexia. These novel and exciting results from TBS in the reading system show that mechanisms derived from the motor system should not be assumed to be applicable to the more complex cognitive networks. If our results are replicated, it will be necessary to reevaluate how we have interpreted past results using cTBS as an inhibitory technique in the language and reading system, particularly as an intervention and rehabilitation technique, and reevaluate how we plan future intervention and rehabilitation studies.

## Data availability statement

The datasets presented in this study can be found in online repositories. The names of the repository/repositories and accession number(s) can be found below: https://osf.io/bewxv/.

## Ethics statement

The studies involving humans were approved by the Georgia State University/Georgia Tech Joint Center for Advanced Brain Imaging Institutional Review Board. The studies were conducted in accordance with the local legislation and institutional requirements. The participants provided their written informed consent to participate in this study.

## Author contributions

RH designed analysis, gathered data, performed analysis, and wrote manuscript. CA designed protocol, gathered data, designed analysis, performed analysis, and edited manuscript. LK designed analysis and edited manuscript. AO and AD gathered data, performed analysis, designed analysis, and edited manuscript. MJ gathered data, performed analysis, and edited manuscript. EB performed analysis and edited manuscript. AW contributed to analysis and edited manuscript. RM oversaw project, designed protocol, designed analysis, and edited manuscript. All authors contributed to the article and approved the submitted version.
